# Development of a Human Breast-Cancer Derived Cell Line Stably Expressing a Bioluminescence Resonance Energy Transfer (BRET)-Based Phosphatidyl Inositol-3 Phosphate (PIP_3_) Biosensor

**DOI:** 10.1371/journal.pone.0092737

**Published:** 2014-03-19

**Authors:** Mei-Shiue Kuo, Johanna Auriau, Cécile Pierre-Eugène, Tarik Issad

**Affiliations:** 1 Institut Cochin, Université Paris Descartes, CNRS (UMR8104), Paris, France; 2 INSERM, U1016, Paris, France; Consiglio Nazionale delle Ricerche (CNR), Italy

## Abstract

Stimulation of tyrosine kinase receptors initiates a signaling cascade that activates PI3K. Activated PI3K uses PIP_2_ to generate PIP_3_, which recruit Akt to the plasma membrane through its pleckstrin homology (PH) domain, permitting its activation by PDKs. Activated Akt controls important biological functions, including cell metabolism, proliferation and survival. The PI3K pathway is therefore an attractive target for drug discovery. However, current assays for measurement of PIP_3_ production are technically demanding and not amenable to high-throughput screening. We have established a MCF-7-derived breast cancer cell line, that stably co-expresses the PH domain of Akt fused to *Renilla* luciferase and YFP fused to a membrane localization signal. This BRET biosensor pair permits to monitor, in real time, in living cells, PIP_3_ production at the plasma membrane upon stimulation by different ligands, including insulin, the insulin analogue glargine, IGF1, IGF2 and EGF. Moreover, several known inhibitors that target different steps of the PI3K/Akt pathway caused inhibition of ligand-induced BRET. Cetuximab, a humanized anti-EGF receptor monoclonal antibody used for the treatment of cancer, completely inhibited EGF-induced BRET, and the tyrosine kinase inhibitor tyrphostine AG1024 inhibited insulin effect on PIP_3_ production. Moreover, the effects of insulin and IGF1 were inhibited by molecules that inhibit PI3K catalytic activity or the interaction between PIP_3_ and the PH domain of Akt. Finally, we showed that human serum induced a dose-dependent increase in BRET signal, suggesting that this stable clone may be used as a prognostic tool to evaluate the PI3K stimulatory activity present in serum of human patients. We have thus established a cell line, suitable for the screening and/or the study of molecules with stimulatory or inhibitory activities on the PI3K/Akt pathway that will constitute a new tool for translational research in diabetes and cancer.

## Introduction

The PI3K (phosphatidylinositol 3-kinase)/Akt pathway regulates multiple biological processes such as metabolism, cell proliferation, survival, migration and apoptosis [1], [2]. It is therefore no surprise that alterations in this pathway have been implicated in the pathogenesis of many human diseases. The serine/threonine kinase Akt/PKB (protein kinase B) belongs to the family of AGC kinases (AMP/GMP kinase and protein kinase C) and consists of three conserved domains, an amino-terminal PH (Pleckstrin homology) domain, a central catalytic domain and a carboxy-terminal regulatory domain. Activation of Akt is a multistep process that is dependent on PI3K activity. The PI3K consists of a p85 regulatory subunit and a p110 catalytic subunit. Upon growth factor stimulation, tyrosine kinase receptors (RTKs) are activated and autophosphorylate on tyrosine residues that serve as docking sites for a number of Src homology 2 (SH2) domain-containing proteins, such as the p85 regulatory subunit of PI3K. p85 can also interact indirectly with RTKs through binding of its SH2 domains to tyrosine phosphorylated residues on adaptor proteins, such as IRSs (Insulin Receptor Substrates). The engagement of p85 to activated receptors induces conformational changes that relieves the intermolecular inhibition of the p110 catalytic subunit and brings it near to its plasma membrane lipid substrate Phosphatidyl Insositol Phosphate 2 (PIP_2_), which is phosphorylated to produce PIP_3_ [3]. PIP_3_ then recruits PDKs (3-phosphoinositide-dependent protein kinases) and Akt to the plasma membrane via their PH domains, where they are subsequently phosphorylated and activated [1], [2]. PTEN (phosphatase and tensin homologue deleted on chromosome 10) terminates the PI3K/Akt signaling by dephosphorylating PIP_3_ into PIP_2_ [4].

**Figure 1:**
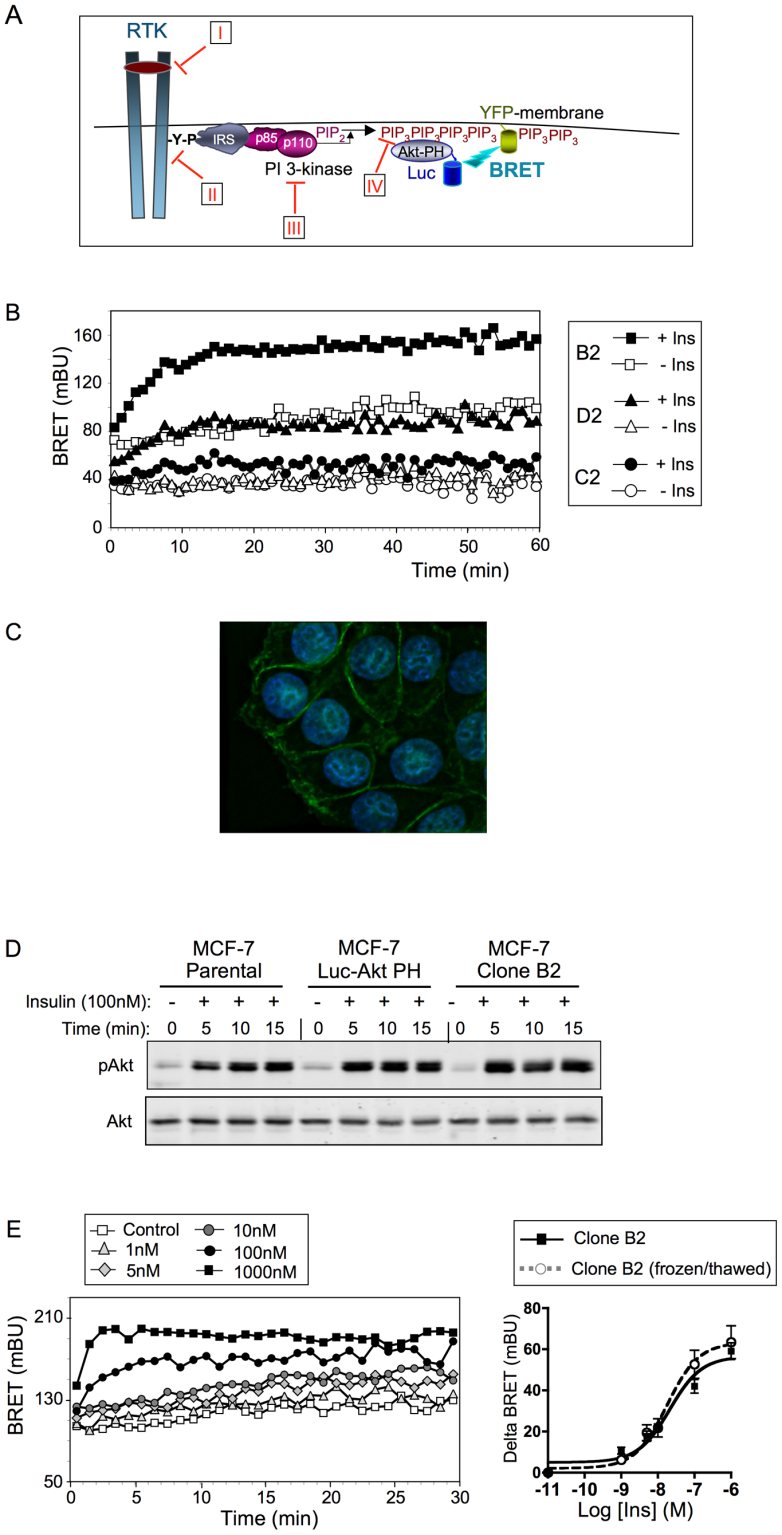
Development of a human breast cancer-derived clone stably expressing a BRET-based PIP_3_ biosensor. (A) Principle of the BRET-based assay used to monitor PIP_3_ production in living cells. Activation of the PI-3 kinase by tyrosine kinase receptors induces phosphorylation of PIP_2_ into PIP_3_ and recruitment of Akt to the plasma membrane through its PH domain. To monitor PIP_3_ production, MCF-7 cells were stably transfected with the PH domain of Akt fused to luciferase (Luc-Akt-PH) and YFP fused to a membrane localization sequence (YFP-membrane). Recruitment of Luc-Akt-PH to the plasma membrane by PIP_3_ results in energy transfer between Luc-Akt-PH and YFP-membrane. This permits to study the pharmacological properties of ligands that activate this pathway, and to evaluate the effects of inhibitory molecules acting on (I) the extracellular part of receptors, (II) the tyrosine kinase activity of the receptors, (III) the catalytic activity of the PI3K, and (IV) the interaction between PIP_3_ and the PH domain of Akt. (B) Transfection of a MCF-7 clone stably expressing Luc-Akt-PH with YFP-membrane cDNA gave rise to 4 sub-clones stably expressing both constructs. Ligand-induced BRET could be detected in only 3 sub-clones (B2, D2, C2). Preliminary BRET experiments indicated that insulin-induced BRET was higher with clone B2. (C) Surface expression of pEYFP-Mem in MCF-7/B2 cells was studied by fluorescence microscopy. YFP fluorescence was detected using a FITC filter and nuclei were visualized using a DAPI filter. The image was obtained by deconvolution analysis. (D) Western-blotting experiment showed that expression of Luc-Akt-PH alone or together with YFP-membrane does not affect insulin-induced phosphorylation of endogenous Akt. (E) Insulin dose-dependently stimulated PIP_3_ production in MCF-7/B2 cells. Left panel: typical real-time experiment showing basal and insulin-stimulated BRET. Right panel: insulin-induced BRET (BRET above basal at the plateau) was determined for each insulin concentration to establish dose-response curves. Results are means ± S.E.M. (standard error of the mean) of 6 to 11 independent experiments. The freeze-thaw cycle did not affect the sensitivity of the cells to insulin stimulation.

The PI3K pathway controls a wide spectrum of important functions, including metabolism, cell growth, proliferation, survival and motility, which, when deregulated, can drive tumor progression. Therefore, this pathway constitutes an attractive target for anti-cancer drug discovery [5]. However, measurement of PIP_3_ production in cells is technically demanding and not easily amenable to high throughput screening assays. Although it has been suggested that recruitment of Akt protein to the plasma membrane could also occur through PIP_3_ independent mechanisms [6], the PH domain of Akt (about 100 amino acids) is highly specific for PIP_3_ and has been previously used, in fusion with a green fluorescent protein, to visualize PIP_3_ production at the plasma membrane using fluorescence microscopy [7], [8]. Using this specific domain, we recently developed a BRET-based assay that permits to monitor, in real time, in living cells, ligand-induced PIP_3_ production at the plasma membrane [9], [10]. In this assay, the yellow fluorescent protein (YFP) is fused with a membrane targeting sequence (YFP-Mem), allowing its addressage at the plasma membrane, and the PH domain of Akt is fused to Renilla Luciferase (Luc-Akt-PH). Upon PIP_3_ production, the recruitment of Luc-Akt-PH to the plasma membrane results in an energy transfer between the luciferase and the YFP (BRET) that can be monitored in living cells cultured in 96 well plates (Fig. 1A). This assay constitutes an excellent tool for the search of molecules that modulate the activity of the PI3K/Akt pathway. In this paper, we took advantage of this simple and robust assay to generate a cell line, derived from human breast cancer MCF (Michigan Cancer Fondation)-7 cells, stably expressing the YFP-Mem and Luc-Akt-PH BRET biosensor pair. We show that this cell line is indeed capable of responding to different growth factors, and that the effects of known inhibitors of the PI3K/Akt pathway can be readily detected, indicating that this stable clone is suitable for screening and/or studying inhibitors or activators of the PI3K/Akt pathway.

## Materials and Methods

### Reagents

Most chemical reagents have been described previously [11], [12]. LY294002 was purchased from Cell Signaling Technology, PIT-1 and DMPIT-1 from Cayman Chemical, and Tyrphostin AG1024 from Santa-Cruz. Growth factors and IGFBP1(Insulin-like growth factor binding protein 1) were from PreproTech. Insulin and Glargine were obtained from the hospital Cochin (AP-HP, Paris). Cetuximab and Trastuzumab were obtained from the hospital Saint-Louis (AP-HP, Paris). Human serum (AB) was from Pel-Freez Biologicals. Centrifugal filter devices for size exclusion of small molecules present in human serum were from Millipore (Microcon 3 kDa).

Anti-Phopho-Akt S473 D9E was from Cell Signaling Technology and anti-Akt H136 from Santa Cruz Biotechnology.

### Expression Vectors

The vector coding for Luc-Akt-PH [9] was obtained using Gateway Technology. Briefly, the Pleckstrin Homology (PH) domain of mouse Akt1 was amplified by PCR and introduced into the pDONR vector according to the manufacturer instruction. The resulting vector was recombined with phRluc-Nter Gateway destination vector (kindly provided by Dr. Patrick Lécine, France) according to the manufacturer instruction.

For selection using hygromycin B resistance, the cDNA coding for YFP-targeted to the plasma membrane (pEYFP-Mem from Clontech) was sub-cloned into pIREShyg3 (Clontech) by using the following primers (5′-CTAGCTAGCATGCTGTGCTGTATGAGAAG-3′ and 5′-GTAGGATCCTT ACTTGTACAGCTCGTCCAT G-3′).

### Generation of Stable Cell Lines

MCF-7 cells were first transfected with the vector Luc-Akt-PH [9]. After cell dilution and geneticin selection (1 mg/ml), we obtained Luc-Akt-PH stable clones. We then generated MCF-7 double stable clonal cells by transfecting one of the Luc-Akt-PH stable clone with the vector pYFP-Mem containing the hygromycin resistance gene. Transfected cells were subsequently diluted to obtain isolated individual cells and then selected in the media containing 1 mg/ml of geneticin plus 200 μg/ml hygromycin B.

### Cell Culture

Parental MCF-7 cells, Luc-Akt-PH stable clonal cells and MCF-7/B2 double stable clonal cells were maintained in Dulbecco’s modified Eagle’s medium supplemented with 4.5 g/l glucose and 10% FBS (fetal bovine serum) [13]. One mg/ml of geneticin were added to the media for Luc-Akt-PH stable cells and 1 mg/ml of geneticin plus 200 μg/ml of hygromycin B were added to the media for double stable clonal cells.

### Western-blotting

MCF-7/B2, MCF-7/Luc-Akt-PH and parental MCF-7 cells were cultured in 6-well plates. After insulin stimulation, cells were lysed in ice-cold buffer containing 50 mM Tris-HCl (pH 8), 137 mM NaCl, 10% (v/v) glycerol, 1% (v/v) NP40, 50 mM NaF, 10 mM di-sodium β-glycerophosphate, 1 mM Na_3_VO_4_, and protease inhibitors (1 μg/ml pepstatin, antipain, leupeptin, aprotinin and AEBSF). Proteins were then analysed by SDS-PAGE (Sodium dodecyl Sulfate-Poly Acrylamide Gel Electrophoresis) followed by western-blotting [14]. Western blots were scanned on the Odyssey Infrared Imaging System (LI-COR Biosciences).

### BRET Assay

The BRET signal has been defined previously as the ratio 530 nm/485 nm obtained when YFP- and Luciferase-fused partners are present, corrected by the ratio 530 nm/485 nm obtained under the same experimental conditions, when only the partner fused to luciferase is present in the assay [15]. For this purpose, in the present study, the MCF-7/Luc-Akt-PH stable clone was cultured in parallel and submitted to the same treatments as the MCF-7/B2 clone. MCF-7/Luc-Akt-PH and MCF-7/B2 stable cells were seeded in 96 wells. Cells were washed with PBS and then incubated for 5 min in PBS in the presence of 2.5 μM coelenterazine. Ligands were then added, and light emission acquisition at 485 nm and 530 nm was started immediately [16], [17]. To study the effects of different inhibitors, cells were first preincubated with inhibitors for 1 h (AG1024, LY294002, Cetuximab, Trastuzimab) or for 4 h (PIT-1 or DMPIT-1). Cells were then washed with PBS, incubated for 5 min in PBS containing 2.5 μM of coelenterazine as well as the inhibitors, and then ligands were added. BRET signal was expressed in milliBRET units (mBU) [15].

### Fluorescence Microscopy

MCF-7/B2 cells were seeded on Lab-Tek™ chamber slide, fixed with 4% paraformaldehyde and the nuclei were stained with DAPI (4′,6-diamidino-2-phenylindole). The subcellular expression of YFP-Mem was visualized by Lecia DMI600 inverted microscope. YFP fluorescence was observed using a FITC (Fluorescein isothiocyanate) filter (excitation: 450–490 nm; emission: 500–550), and the nuclei were visualized using a DAPI filter (excitation: 340–380 nm; emission: 450–490 nm). Images were obtained by deconvolution analysis.

### Proliferation Assay by YFP Fluorescence Measurement

All measurements of YFP fluorescence (excitation at 480 nm and emission at 532 nm) were performed at room temperature using Typhoon Laser scanner (GE Healthcare). MCF-7/Luc-Akt-PH and MCF-7/B2 cells were seeded in 96-well microplates at a density of 15000 cells per well. The first fluorescent measurement was performed on the next day (Day 0) and culture media were then replaced by fresh media containing only 0.1% FBS. Cells were then cultured for 48 h in absence or presence of 1 mM insulin and various inhibitors (high insulin concentrations were used in proliferation assays, in order to compensate for insulin degradation that may occur during long-term incubations (24 or 48 h) in medium containing low amount of serum). Fluorescent measurements were carried out after 24 h (Day 1) and 48 h (Day 2). Each experimental condition was performed in triplicate. Specific YFP fluorescence of MCF-7/B2 cells was obtained by substracting the fluorescence obtained with the MCF-7/Luc-Akt-PH cells cultured in parallel under the same experimental conditions (background fluorescence). The effects of treatments on cell proliferation were obtained by dividing the specific YFP fluorescence measured on the day of the assay by the initial fluorescence (specific YFP fluorescence measured at Day 0).

### Statistical Analysis

Statistical analyses were performed by ANOVA (Analysis of variance) followed by Tukey’s test using Prism Software (GraphPad, San Diego, CA, USA).

## Results

### Generation of Stable Clonal Cell Lines

MCF-7 cells were transfected with Luc-Akt-PH cDNA, and after geneticin selection, a clone stably expressing Luc-Akt-PH was used for establishing the double transfected cell line. Double clonal cell lines (MCF-7 cells stably expressing Luc-AKT-PH and YFP-Mem) were obtained from this stable clone by transfection with the vector pEYFP-Mem bearing hygromycin B resistance. After selection in the presence of both geneticin and hygromycin B, four clones expressing both constructs were obtained. One clone was not responsive to insulin (not shown), whereas in the three other clones, increased BRET was observed upon insulin stimulation, with clone B2 being the most responsive (Fig. 1B). Fluorescent microscopy showed that YFP-Mem was indeed correctly targeted at the plasma membrane in this clone (Fig. 1C). To verify that expression of Luc-Akt-PH and YFP-Mem did not alter the activity of the PI3K/Akt signaling pathway, we compared Akt phosphorylation in Luc-Akt-PH and B2 clones with the parental MCF-7 cell line upon insulin stimulation. Western-blotting experiments with anti-phosphoAkt antibody showed that similar levels of phosphorylation were obtained after 5, 10 and 15 min of stimulation with 100 nM insulin (Fig. 1D).

To ensure that the B2 clone can be stored frozen and submitted to freeze/thaw cycles without losing its reporter properties, we evaluated insulin responsiveness of the cells before and after several weeks of storage in liquid nitrogen (Fig. 1E). We observed that insulin dose-dependently stimulated BRET. A dose-response curve (established using insulin-induced BRET values at the plateau) indicated that insulin stimulated PIP_3_ production with a half maximal effective concentration (EC50) of 20,18±7.93 nM. After several weeks in liquid nitrogen, the clone was thawed out, amplified in 75 cm^2^ flasks and then plated in 96 well plates for reassessment of insulin responsiveness. The new dose-response curve was perfectly super-imposable to the one obtained prior to the freeze/thaw cycle, and the EC50s of insulin remained identical (EC50 of 20.18±7.93 nM before and 18.76±4.85 nM after freezing/thawing, n = 6 to 11). This indicated that a freeze/thaw cycle of B2 cells did not change their response to insulin. We thus chose clone B2 for further characterization.

### Ligand-induced PIP_3_ Production in MCF-7/B2 Clone

**Figure 2:**
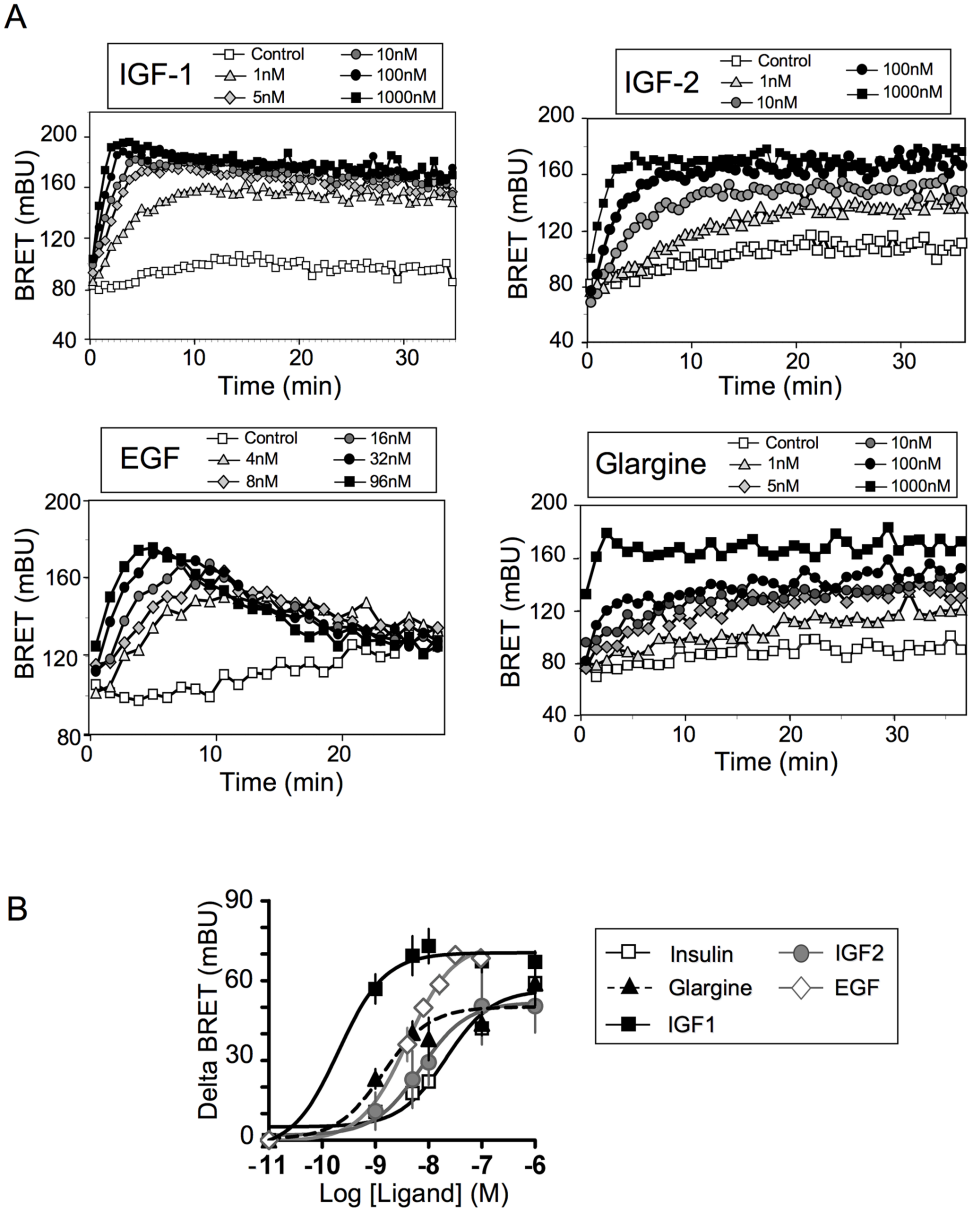
Effect of IGF1, IGF2, EGF and glargine on PIP_3_ production in MCF-7/B2 cells. (A) Typical experiments showing real-time effects of IGF1, IGF2, EGF and glargine on PIP_3_ production in MCF-7/B2 cells. (B) Dose-dependent effect of IGF1, IGF2, EGF, glargine and insulin on PIP_3_ production in MCF-7/B2 cells. Ligand-induced BRET (BRET above basal) at the plateau (IGF1, IGF2, Insulin, Glargine) or at the peak (EGF) was determined for each ligand concentration and was used to establish dose-response curves. Results are means ± S.E.M. of 3 to 8 independent experiments. EC50 for insulin, IGF1, IGF2, EGF and glargine are given in the result section.

We then evaluated the effects of IGF1 (Insulin-like growth factor 1), IGF2 (Insulin-like growth factor 2), EGF (Epidermal Growth Factor) and glargine (an insulin analogue used in the treatment of diabetes) on PIP_3_ production in the MCF-7/B2 clone. We observed that these ligands increased BRET signal in a dose-dependent manner (Fig. 2A). Interestingly, whereas IGF1, IGF2 and glargine-induced signals reached a plateau and remained stable during the course of the experiment, EGF produced a transient increase in BRET signal, which returned to basal levels within 20 min after stimulation. Dose-response curves were established (Fig. 2B) using ligand-induced BRET (BRET above basal at the plateau for IGF1, IGF2 and glargine, or at the peak value for EGF). IGF1 (EC50 of 0.34±0.05 nM, n = 6 to 8) stimulated PIP_3_ production much more efficiently than insulin (EC50 of 20.18±7.93 nM, n = 11, p<0.001). IGF1 was also more efficient than IGF2 (EC50 of 16.75±10.01 nM, n = 4, p<0.01). The EC50 of EGF (4.09±0.88 nM, n = 3) was intermediary between that of IGF-1 and IGF2. Glargine, *in vitro*, is known to activate IGF1R (IGF1 receptor) and Insulin/IGF1 hybrid receptors with higher efficiency than insulin [9], [18]. We observed that glargine (EC50 of 1.98±0.87 nM) stimulated PIP_3_ production much more efficiently than insulin in B2 cells (n = 4, p<0.01). In a previous study, we measured EC50 for stimulation of PIP_3_ production by IGF1, insulin and glargine in the parental MCF-7 cells transiently expressing the Luc-Akt-PH/YFP-Mem biosensor pair [9]. The EC50s obtained in the present study are in full agreement with those obtained in our previous study, indicating that the pharmacological properties of the parental cell line are preserved in MCF-7/B2 clone.

### Effect of Inhibitors of the RTK/PI-3 Kinase Pathway on BRET in MCF-7/B2 Cells

We then investigated whether the MCF-7/B2 clone could be used to study the effects of inhibitors that target the PI3K pathway at different levels (Fig. 1).

**Figure 3:**
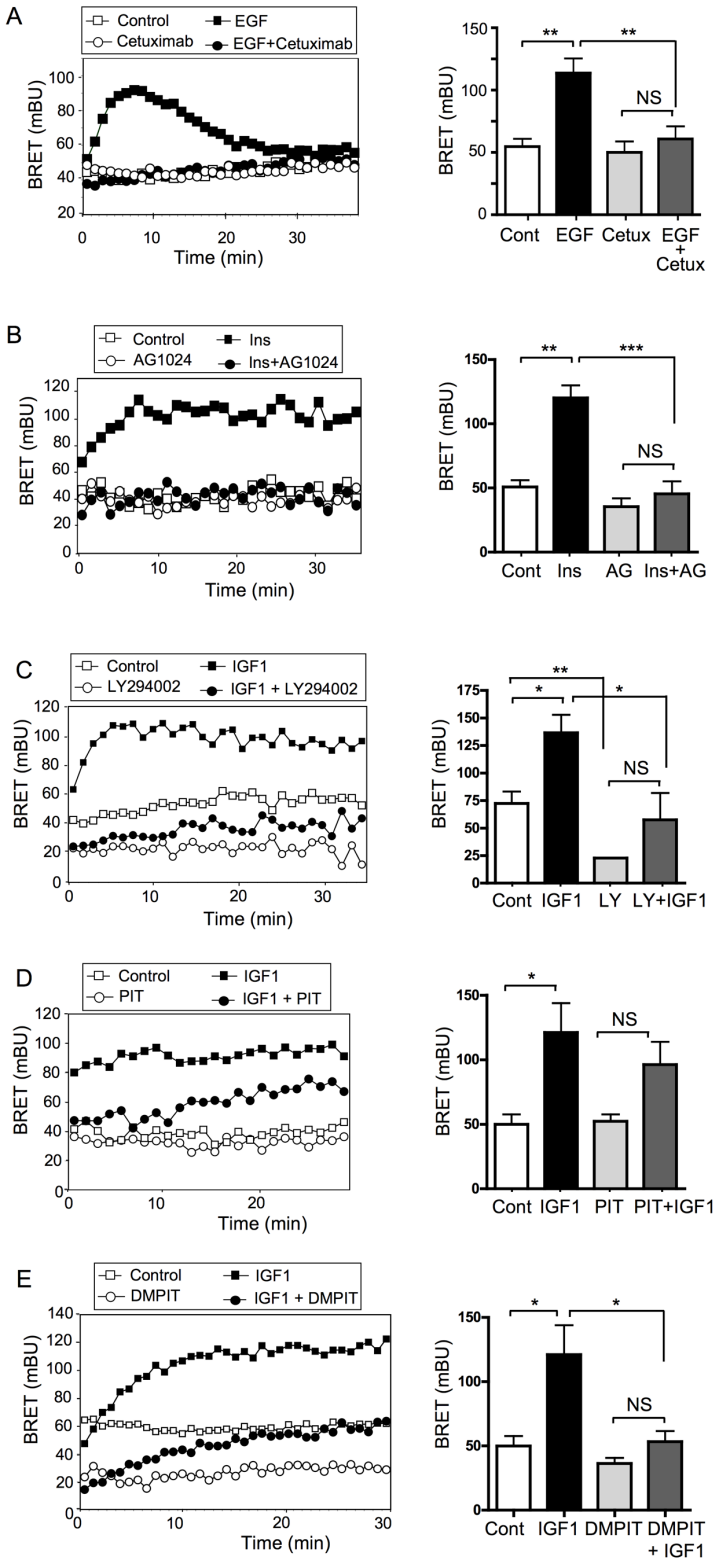
Effect of inhibitors of the PI3K/Akt signaling pathway on BRET signal in MCF-7/B2 cells. (A) MCF-7/B2 cells were preincubated for 1 h in absence or presence of 20 ng/μl of the humanized anti-EGFR antibody Cetuximab. Cells were then stimulated with EGF (32 nM), and light emission acquisition started immediately. A typical real-time BRET experiment (left panel) and the mean ± SEM of BRET values at the peak (right panel) are shown (n = 3). (B) MCF-7/B2 cells were preincubated for 1 h in absence or presence of 25 μM of the tyrphostin AG1024. Cells were then stimulated with 100 nM insulin, and light emission acquisition started immediately. A typical real-time BRET experiment (left panel) and the mean ± SEM of BRET values at the plateau (right panel) are shown (n = 3). (C) MCF-7/B2 cells were preincubated for 1 h in absence or presence of 25 μM of the PI3K inhibitor LY294002. Cells were then stimulated with 10 nM IGF1 and light emission acquisition started immediately. A typical real-time BRET experiments (left panel) and the mean ± SEM of BRET values at the plateau (right panel) of 3 independent experiments are shown. (D, E) MCF-7/B2 cells were preincubated for 4 h in absence or presence of 10 μM of the inhibitors of Akt-PH/PIP_3_ interaction PIT-1 (D) and DMPIT-1 (E). Cells were then stimulated with 10 nM IGF1, and light emission acquisition started immediately. Typical real-time BRET experiments (left panels) and the mean ± SEM of BRET values at the plateau (right panels) of 3 to 4 independent experiments are shown. Statistical analysis was performed using ANOVA followed by Tukey’s test. *, P<0.05; **, P<0.01; ***, P<0.001; NS, Non significant.

We firstly studied the effect of Cetuximab, a chimeric humanized antibody that inhibits EGFR (EGF receptor) [19]. We observed that whereas Cetuximab had no effect on basal BRET signal, it completely abrogated the effect of EGF on PIP_3_ production (Fig. 3A). The specificity of this inhibitory effect was demonstrated by the absence of inhibition of IGF-1-induced BRET by Cetuximab (Supplementary Fig. S1A). Moreover, Trastuzumab, which specifically inhibits HER2 (Human epidermal growth factor receptor 2) [20], had no effect on basal or EGF-induced BRET in these cells (Supplementary Fig. S1B), in agreement with the HER2 negative phenotype of MCF-7 cells [21].

The Tyrphostin AG1024 is a protein tyrosine kinase inhibitor that selectively inhibits IR (insulin receptor) and IGF1R [22], [23]. Whereas AG1024 had no significant effect on basal-BRET, it completely inhibited insulin-induced BRET signal (Fig. 3B). Therefore, inhibitors of RTK induce an inhibition of PIP_3_ production that can be detected using MCF-7/B2 cells.

We also evaluated the effect of LY294002, a known inhibitor of the catalytic activity of PI3K [24], on basal, insulin, and IGF1-stimulated BRET signal. We observed that LY294002 significantly inhibited basal (p<0.01) and ligand-induced (p<0.05) PIP_3_ production (Fig. 3C and Supplementary Fig. S2A). Recent studies indicated PI3K/Akt signaling pathway can be inhibited by small non-phosphoinositide molecules that disrupt the interaction between PIP_3_ and the PH-domain of Akt [25]. Binding of these molecules to Akt-PH domain impairs Akt recruitment to the plasma membrane and its activation by PDKs. We evaluated whether the inhibitory effects of two non-phosphoinositide PIP_3_ inhibitors, PIT-1 and DMPIT-1, could be detected in the MCF-7/B2 clone. PIT-1 had no effect on basal BRET signal and only marginal effects on IGF1 (Fig. 3D) and insulin-stimulated (Supplementary Fig. S2B) BRET signals. DMPIT-1 significantly (p<0.05) inhibited IGF-1 (Fig. 3E) and insulin-stimulated (Supplementary Fig. S2C) BRET signals. The higher inhibitory effect of DMPIT-1 compared to PIT-1 on this signaling pathway has been described previously and probably reflects its higher cell permeability [25].

### The Effect of Inhibitors on MCF-7/B2 Cell Proliferation can be Directly Evaluated by Measuring YFP Fluorescence

**Figure 4:**
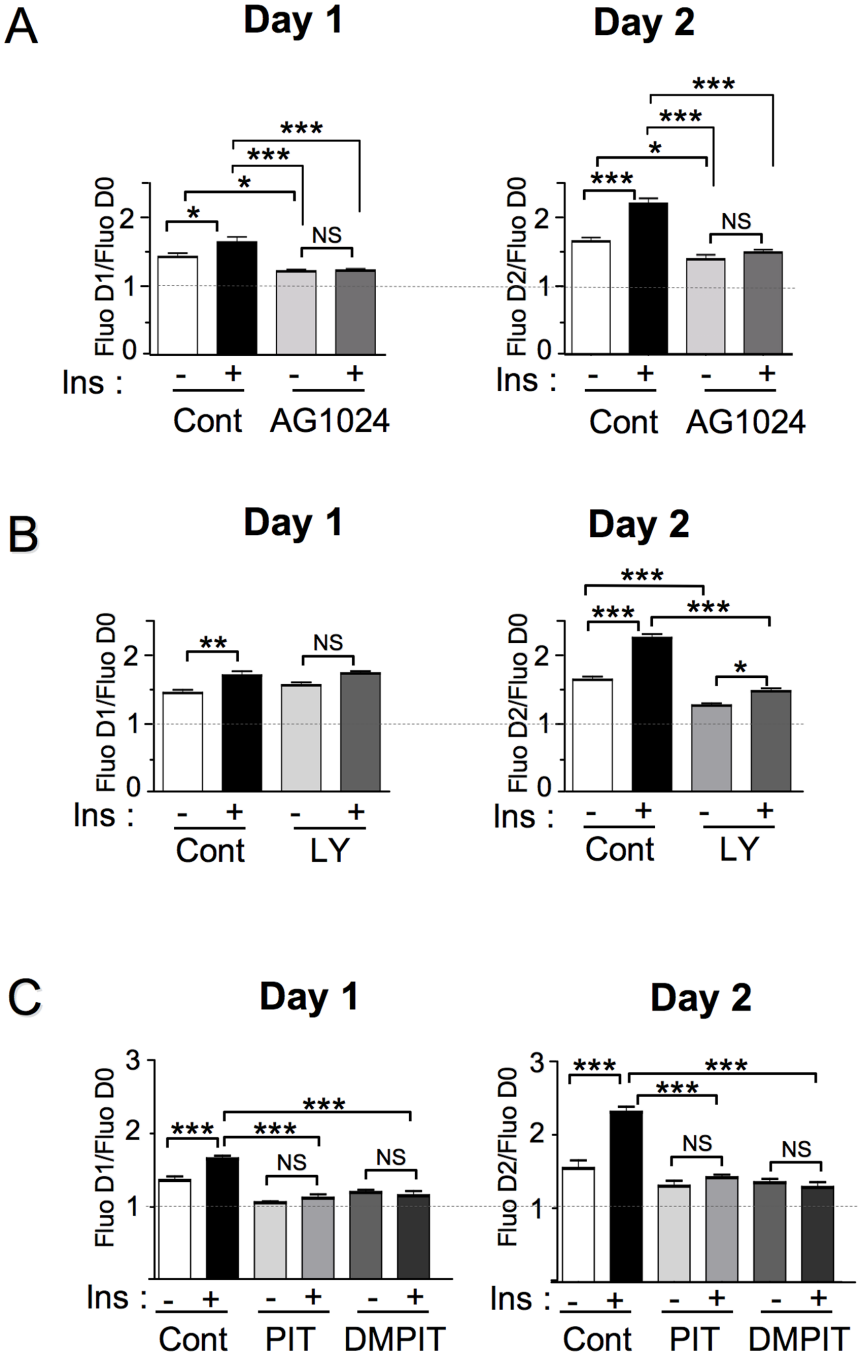
The effects of treatments on MCF-7/B2 cell proliferation can be monitored by YFP fluorescence measurement. MCF-7/B2 cells were plated in 96 well plates at a density of 15000 cells/well. 24 h after plating, YFP fluorescence in each well was measured at 530 nm after excitation at 480 nm (D0). Cells were then treated with ligands and/or inhibitors, and fluorescence measurements were performed 24 h (D1) and 48 h (D2) later using a Typhoon Laser scanner. The effects of treatments on cell proliferation were estimated by dividing the fluorescence after treatment by initial fluorescence. Background fluorescence of the cells was obtained by culturing in parallel MCF-7/Luc-Akt-PH cells under the same experimental conditions. (A) Cells were cultured in presence of 1 μM of insulin in absence or presence of the 25 μM of AG1024 (B) Cells were cultured in presence of 1 μM of insulin in absence or presence of 50 μM of the PI3K inhibitor LY294002 (C) Cells were cultured in presence of 1 μM of insulin in absence or presence of 10 μM of the inhibitors of Akt-PH/PIP_3_ interaction PIT-1 and DMPIT-1. Mean ± SEM of fluorescence ratios of 3 to 4 independent experiments are shown. Statistical analysis was performed using ANOVA followed by Tukey’s test. *, P<0.05; **, P<0.01; ***, P<0.001; NS, Non significant.

Since MCF-7/B2 cells stably express YFP, we evaluated whether the effects of inhibitors of the PI3K pathway on cell proliferation could be monitored in parallel by a simple measurement of the YFP fluorescence. We first evaluated the effect of the tyrphostin AG1024 on cell proliferation. As shown in Figure 4A, insulin induced an increase in cell proliferation, resulting in a significant increase in fluorescence at Day 1 (p<0.05) and a more marked increase at Day 2 (p<0.001). AG1024 treatment significantly reduced basal cell proliferation (p<0.05), and completely abrogated (p<0.001) the stimulatory effect of insulin on proliferation (Fig. 4A).

The PI3K inhibitor LY294002 also inhibited insulin-induced cell proliferation (Fig. 4B), and this effect was more readily observed at Day 2 (p<0.001).

We also evaluated the effect of PIT-1 and DMPIT-1, and found that both compounds significantly inhibited (p<0.001) insulin-induced cell proliferation (Fig. 4C).

These results support the idea that the biological activity of molecules that modulate PIP_3_ signaling pathway can be evaluated in parallel using a simple fluorescent measurement to monitor their effects on cell proliferation.

### The B2 Clone can be used to Evaluate the Biological Activity of Human Serum

**Figure 5:**
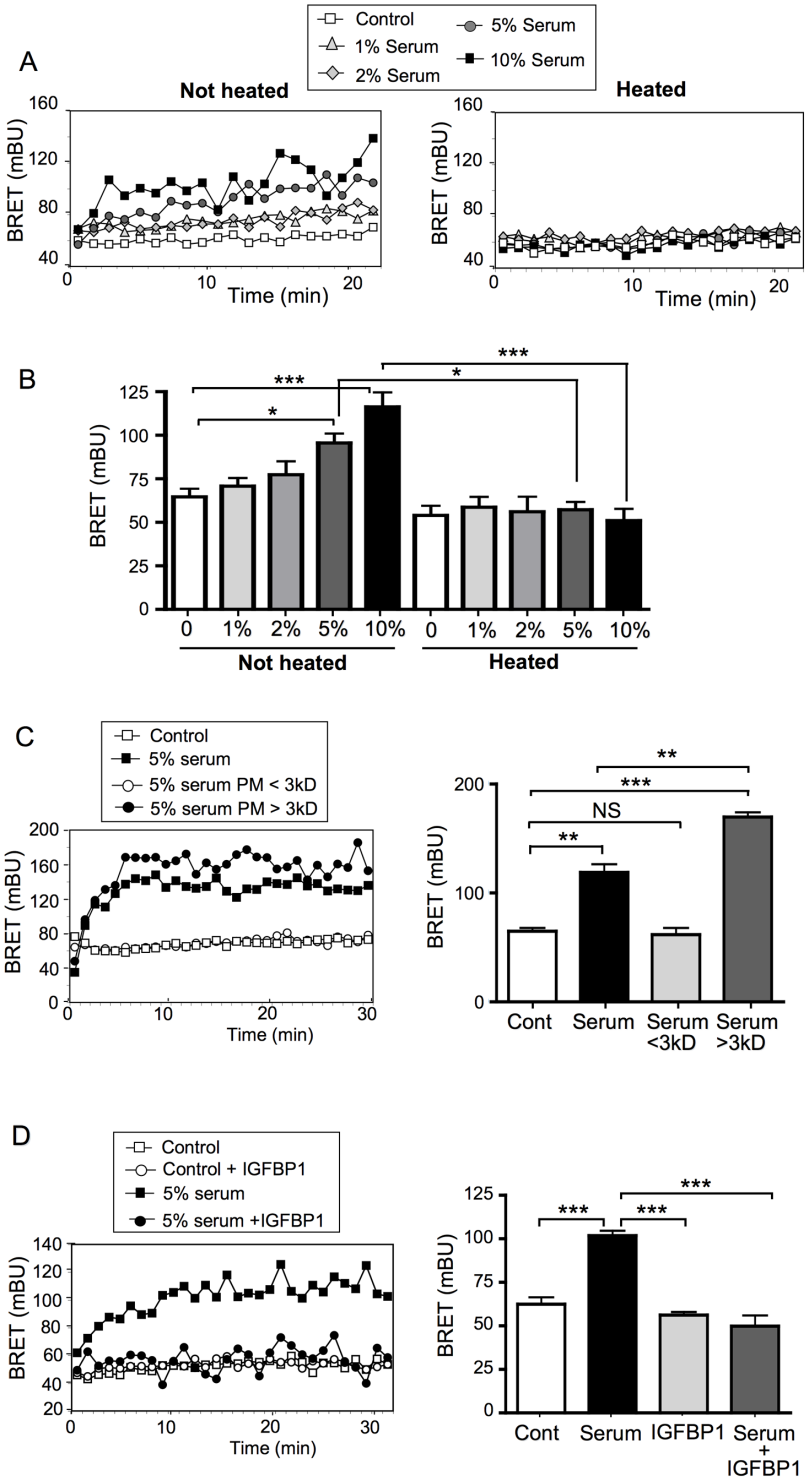
The PI3K stimulatory activity present in human serum can be evaluated using the MCF-7/B2 cells. (A, B) MCF-7/B2 cells were starved overnight in culture medium containing only 0.1% FBS. Cells were then stimulated with PBS containing 0%, 1%, 2%, 5% or 10% human serum that had been previously heated or not at 95°C during 1 h, and light emission acquisition started immediately. (A) A typical real-time BRET experiment is shown. (B) Means ± SEM of BRET values at the plateau of 3 to 7 independent experiments are shown. (C) MCF-7/B2 cells were starved overnight in culture medium containing only 0.1% FBS. Cells were then stimulated with 5% human serum previously submitted or not to centrifugation on a centrifugal filter device with a molecular weight cut-off of 3 kDa. A typical real-time BRET experiment (left panel) and means ± SEM of BRET values at the plateau (right panel) are shown (n = 3). (D) MCF-7/B2 cells were starved overnight in culture medium containing only 0.1% FBS. Cells were then stimulated with 5% human serum that had been pre-incubated for 1 h in presence of 50 μM IGFBP1. A typical real-time BRET experiment (left panel) and means ± SEM of BRET values at the plateau (right panel) are shown (n = 3). Statistical analysis was performed using ANOVA followed by Tukey’s test. *, P<0.05; **, P<0.01; ***, P<0.001; NS, Non significant.

Human serum contains several molecules and growth factors that can activate the PI3K pathway. We therefore tested whether the MCF-7/B2 cell line could be used to monitor PI3K stimulatory activity potentially present in human serum. MCF-7/B2 cells were cultured overnight in medium containing only 0.1% FBS. Cells were then stimulated with PBS containing 1 to 10% of commercially available human serum. As shown in Figure 5A and B, human serum can induce PIP_3_ production in a dose-dependent manner. Heating the serum at 95°C for 1 h completely abolished its ability to stimulate PIP_3_ production, indicating that the active molecules are destroyed by the heat. In order to determine whether the active factors present in serum corresponded to small molecules such as metabolites, steroid hormones or small peptides, we centrifuged human serum on a centrifugal filter device with a molecular weight cut-off of 3 kDa, and evaluated the PIP_3_ stimulatory activity present in the low- and high-molecular weight fractions (filtrate and retentate fractions, respectively). We observed that the low (<3 kDa) molecular weight fraction had no effect on PIP_3_ production, whereas the PIP_3_-stimulatory activity was present in the high (>3 kDa) molecular weight fraction (Fig. 5C). Moreover, the stimulatory activity in the retentate was significantly higher (p<0.01) than the initial activity present in non-filtrated serum, suggesting that some small molecules with inhibitory activity may have been lost during filtration.

IGF1 and IGF2 are known to be strong stimulators of MCF-7 cell growth, and our results demonstrated that these growth factors stimulated PIP_3_ production very efficiently in the MCF-7/B2 cell line (Fig. 2). We therefore hypothesized that IGF1 and/or IGF2 could be the active factors present in human serum. IGFBP1 (Insulin-like growth factor binding protein-1) is a secreted protein that binds to and inhibits the stimulatory activity of IGF1 and IGF2 on IGF-1R [26], [27]. To determine whether the PI3K stimulatory activity present in serum was mediated by IGFs, the serum was pre-incubated with recombinant IGFBP1 for 1 h prior to stimulation of MCF-7/B2 cells. IGFBP1 alone had no effect on PIP_3_ production in B2 cells, whereas it completely inhibited the stimulatory effect of 5% serum (Fig. 5C). As shown in Supplementary Figure S3, the inhibitory effect of IGFBP1 was dose-dependent, with a maximal inhibitory activity at 50 μM IGFBP1. Together, these results demonstrate that the MCF-7/B2 clone can be used to evaluate PIP_3_ stimulatory activity present in human serum, and that in the commercial batch of human serum used in this study, this stimulatory effect is mediated by a heat-sensitive molecule with a molecular weight higher than 3 kDa, the activity of which is inhibited by the presence of IGFBP1, suggesting that IGF1 and/or IGF2 could be the factors responsible for activation of the PI3K pathway.

## Discussion

Deregulation of the PI3K pathway has been implicated in many human diseases. For instance, hyperactivation and deficiency of this pathway are associated with the development of cancer and diabetes, respectively. The PI3K pathway is thus a very attractive target for drug screening. For the development of molecules that modulate this pathway, it is important to have a simple, fast and robust assay to measure PI3K activation. Typical methods to measure PI3K activity involve extraction of radioactive products after ^32^P labeling of phosphoinositide substrates and subsequent separation using thin-layer chromatography or High-performance liquid chromatography (HPLC) [28]. These highly time-consuming and costly assays produce radioactive waste and are not suitable for high-throughput screening of molecules. More recently, PI3K activity assays based on ELISA have been developed [29], eliminating the need of radioactivity and thin layer chromatography or HPLC steps. However, these assays remain time-consuming, as they involve extraction, incubation, separation and washing steps. In contrast to these non-homogenous and time-consuming methods, the stable cell line that we described here provides a simple and robust alternative for the study of molecules and ligands that modulate the PI3K pathway. Indeed, we showed that the activation of the PI3K pathway by insulin and growth factors can be monitored in real time, and pharmacological profiles of activation by endogenously expressed receptors could be established for each ligand in dose-response experiments. Whereas both EGFR and IGF1R display high (sub-namolar) affinity for their respective ligands, MCF-7 cells express much lower levels EGFR than IGF1R (3000 EGFR/cell versus 20000 to 100000 IGF1R/cell) [30]–[34]. Although IR are also highly expressed in MCF-7 cells (up to 100000 IR/cell [35]), a large proportion of IR are engaged in IR/IGF1R hybrids [9], [36], which display much higher affinity for IGF1 than for insulin [9], [37]–[39]. Thus, the pharmacological profiles for PIP_3_ production induced by the different ligands used in the present study are in agreement with expression levels and pharmacological properties of their cognate receptors. Indeed, a study of the effect of these ligands on proliferation of MCF-7 cells showed similar order of potency of the different ligands (IGF1>EGF>IGF2>Insulin) [40]. Moreover, in a more recent study using non-transfected MCF-7 cells [9], EC50s of insulin, IGF1 and Glargine for Akt phosphorylation, measured by in-cell western, were found to be very similar to EC50s of these ligands for PIP_3_ production in MCF-7/B2 cells.

Interestingly, EGF-induced PIP_3_ production was transient, reaching a peak between 5 and 10 min after stimulation. In agreement with this observation, several studies have shown, in different cell models, that upon EGF stimulation, activation of EGFR and its downstream signaling pathways, including PI3K/Akt pathway, are transient, due to rapid internalization and down-regulation of the receptor as well as to negative feedback loops in the signaling pathways [28], [41]–[44]. Although performing time-course and dose-response experiments using classical methods for PIP_3_ production measurements is technically highly demanding, one study, using rabbit corneal epithelial cells metabolically labeled with ^32^P-phosphate, showed that EGF-stimulated PIP_3_ production was transient, peaking at about 5 min of stimulation, with an EC50 in the nanomolar range [28]. Therefore, these kinetics and pharmacological profile are very similar to those obtained for EGF-induced PIP_3_ production measured by BRET in MCF-7/B2 cells.

We also showed that our cell line permits the detection of the inhibitory effects of molecules acting on the extra-cellular part of receptors, on their tyrosine kinase activities, on the catalytic activity of PI3K or on the interaction between PIP_3_ and the PH domain of Akt. Therefore, the MCF-7/B2 clone can be used for the identification and/or analyses of activators or inhibitors of the PI3K pathway acting at different levels, from ligand binding to Akt recruitment to the membrane. More specifically, we demonstrated that the inhibitory effect of a humanized monoclonal antibody, such as Cetuximab (which is used for the treatment of human cancers [45]) can be studied using MCF-7/B2 cells. Allosteric inhibitors of Akt that interfere with its binding to PIP_3_, are also being developed for the treatment of PI3K dependent cancers [46]–[48]. As exemplified by the study of the effects of PIT-1 and DMPIT-1, the efficiency of such inhibitors can be evaluated using MCF-7/B2 cells.

In addition, we showed that the stable expression of YFP in these cells permits to monitor cell proliferation by a simple fluorescent measurement after 24 or 48 h of treatment. Therefore, the stimulatory or inhibitory effects of molecules on cell proliferation can be evaluated in parallel in 96 well plates. However, differences between BRET and proliferation may also be observed. Thus, whereas PIT-1 displayed lower efficiency compared to DMPIT-1 in BRET experiments, both compounds showed similar inhibitory effect on cell proliferation. This suggests that differences in cell permeability between PIT-1 and DMPIT-1 [25] may impact their efficiency for short-term treatments (4 h incubations in BRET experiments) but not for long-term treatments (24 h–48 h incubations in proliferation experiments).

Although we have not examined in the present work the potential usefulness of this cell line for *in vivo* applications, the stable expression of luciferase should also permit to follow, by charged-coupled device camera imaging, the effects of *in vitro* identified molecules on tumor growth *in vivo*, after implantation of MCF-7/B2 cells in immunodeficient mice.

Finally, this tool should allow the study of PI3K-stimulatory activity present in biological fluids. Indeed, we showed that MCF-7/B2 clone could be used to evaluate the effect of human serum on PIP_3_ production. Serum is likely to contain various molecules capable of stimulating or inhibiting the PI3K pathway. We observed that a commercially available batch of human serum stimulated PIP_3_ production in a dose-dependent manner. Interestingly, the stimulatory activity appeared to be heat-sensitive and was due to molecules with molecular weight higher than 3 kDa, as this activity was lost in the low molecular weight fraction obtained after centrifugation on a 3 kDa-cut-off size exclusion filtration membrane.

In human serum, the biological activities of hormones, growth factors and cytokines can be regulated by circulating binding proteins [49]–[53]. More specifically, IGFBPs have been shown to regulate the biological activity of IGFs [54]. Interestingly, we observed that the stimulatory effect of human serum could be completely abrogated by pre-incubation of the serum with recombinant human IGFBP1. This suggests that IGFs may be the principal molecules present in the batch of serum used in this study that activate PI3K signaling. Various studies have shown that high circulating levels of IGFs are associated with an increased risk of several cancers [50]. The bioavailability of IGFs is modulated by the IGFBP family that contains six structurally similar proteins with high affinity for IGFs. Functioning as IGFs carriers within the circulation, IGFBPs regulate IGFs action by inhibiting or facilitating the binding of IGFs to IGF1R. IGFBPs can inhibit IGFs action by sequestering IGFs away from IGF1R. However, the interaction between IGFBPs and matrix components may concentrate IGFs near their receptor, thus enhancing IGFs activity [54], [55]. IGFBPs proteolysis by IGFBPs-specific proteases represents an essential mechanism for IGF release [50], [54], [55]. Therefore, the biological activity of IGFs depends not only on their concentration in the serum, but also on the relative amount of IGFBPs and IGFBP-specific proteases [56], [57]. Since the PI3K-stimulatory activity present in human serum can be directly evaluated using MCF-7/B2 cells, it could be envisaged to use this cell line as a prognostic tool to predict the proliferative potential of sera from patients. Obviously, additional studies, using sera from different origins and pathologies, will be needed to establish the value of MCF-7/B2 cells as a prognostic tool to evaluate the proliferative activity present in the serum of patients.

MCF-7/B2 cells could also be of interest for evaluating the consequence of hormonal or pharmacological treatments on modulation of PI3K-stimulatory activity in serum. For instance, glargine, a long-lasting insulin analogue used for the treatment of diabetes, displays higher mitogenic properties on culture cells *in vitro*, when compared to insulin. However, when injected *in vivo*, glargine is rapidly converted into metabolites [58], [59], [60] with pharmacological properties similar to insulin [9], [18]. In order to determine the consequences of glargine treatment on the proliferative activity present in human serum, attempts have been made to compare the effects of sera from insulin and glargine-treated diabetic patients on the proliferation of MCF-7 cells [61]. However, proliferation assays on cultured cells are time consuming (72 hours of cell culture in the above mentioned study), and the number of cases evaluated remained very small (31 patients in total), considerably limiting the clinical significance of this study. In contrast, with MCF-7/B2 cells, the read-out (PIP_3_ production) can be obtained very rapidly (BRET plateau reached after 10–15 minutes of incubation with 5% serum). Therefore, our cell line should constitute an excellent tool to evaluate in clinical assays the PI3K stimulatory activity present in sera from large cohorts of patients treated with different insulin analogues or other medications. Finally, MCF-7/B2 cells could also be a useful tool to study PIP_3_-stimulatory activity present in conditioned media from normal or cancer cells, in order to evaluate whether proteins or small molecules secreted by these cells may affect the PI-3 kinase pathway through autocrine or paracrine mechanisms [27].

In summary, we have developed a human breast cancer cell line expressing a BRET-based biosensor to monitor the PI3K/Akt pathway. This cell line constitutes a powerful tool for the study of activators and inhibitors acting at different levels on this signaling pathway. MCF-7/B2 cells could also be used in high-throughput screening assays for the identification of new molecules that modulate the PI3K/Akt pathway. Indeed, after seeding in 96 wells plates, cells are ready to use for BRET experiments on the following day. Moreover, taking advantage of the stable expression of YFP, these cells can also be used to monitor the effect of different treatments on cell proliferation by simple fluorescent measurements. Finally, MCF-7/B2 cells can also be used for evaluating the PI3K activity present in human serum.

## Supporting Information

Figure S1 **Effects of anti-EGFR and anti-HER2 antibodies on IGF1 and EGF-stimulated PIP_3_ production.** (A) MCF-7/B2 cells were preincubated for 1 h in absence or presence of 20 ng/μl of the humanized anti-EGFR antibody Cetuximab. Cells were then stimulated with IGF1 (10 nM), and light emission acquisition started immediately. A typical real-time BRET experiment (left panel) and the mean ± SEM of BRET values at the plateau (right panel) are shown (n = 3). (B) MCF-7/B2 cells were preincubated for 1 h in absence or presence of 20 ng/μl of the humanized anti-HER2 antibody Trastuzumab. Cells were then stimulated with EGF (32 nM), and light emission acquisition started immediately. A typical real-time BRET experiment (left panel) and the mean ± SEM of BRET values at the peak (right panel) are shown (n = 3). Statistical analysis was performed using ANOVA followed by Tukey’s test. **, P<0.01; ***, P<0.001; NS, Non significant.
(PDF)

Figure S2 **Effect of inhibitors of PI3K signaling on insulin-stimulated PIP_3_ production in MCF-7/B2 cells.** (A) MCF-7/B2 cells were preincubated for 1 h in absence or presence of 25 μM of the PI3K inhibitor LY294002. Cells were then stimulated with 10 nM insulin and light emission acquisition started immediately. A typical real-time BRET experiment (left panel) and the mean ± SEM of BRET values at the plateau (right panel) of 4 independent experiments are shown. (B, C) MCF-7/B2 cells were preincubated for 4 h in absence or presence of 10 μM of the inhibitors of Akt-PH/PIP_3_ interaction PIT-1 (B) and DMPIT-1 (C). Cells were then stimulated with 10 nM insulin, and light emission acquisition started immediately. Typical real-time BRET experiments (left panels) and mean ± SEM of BRET values at the plateau (right panels) of 3 to 5 independent experiments are shown. Statistical analysis was performed using ANOVA followed by Tukey’s test. *, P<0.05; **, P<0.01; ***, P<0.001; NS, Non significant.
(PDF)

Figure S3 **Dose-dependent effect of IGFBP1 on human serum induced PIP_3_ production in MCF-7/B2 cells.** MCF-7/B2 cells were starved overnight in culture medium containing only 0.1% FBS. Cells were then stimulated with 5% human serum that had been pre-incubated for 1 h in presence of increasing concentrations of IGFBP1. Means ± SEM of BRET values at the plateau of 4 to 7 independent experiments are shown. Statistical analysis was performed using ANOVA followed by Tukey’s test. *, P<0.05; **, P<0.01; ***, P<0.001.
(PDF)
