# Supplementary material for: Development of a Human Breast-Cancer Derived Cell Line Stably Expressing a Bioluminescence Resonance Energy Transfer (BRET)-Based Phosphatidyl Inositol-3 Phosphate (PIP3) Biosensor
Source: PLoS One. 2014 Mar 19;9(3):e92737. doi: 10.1371/journal.pone.0092737 (PMC3960261; doi:10.1371/journal.pone.0092737)
Supplement: Figure S1 — Effects of anti-EGFR and anti-HER2 antibodies on IGF1 and EGF-stimulated PIP3 production. (A) MCF-7/B2 cells were preincubated for 1 h in absence or presence of 20 ng/μl of the humanized anti-EGFR antibody Cetuximab. Cells were then stimulated with IGF1 (10 nM), and light emission acquisition started immediately. A typical real-time BRET experiment (left panel) and the mean ± SEM of BRET values at the plateau (right panel) are shown (n = 3). (B) MCF-7/B2 cells were preincubated for 1 h in absence or presence of 20 ng/μl of the humanized anti-HER2 antibody Trastuzumab. Cells were then stimulated with EGF (32 nM), and light emission acquisition started immediately. A typical real-time BRET experiment (left panel) and the mean ± SEM of BRET values at the peak (right panel) are shown (n = 3). Statistical analysis was performed using ANOVA followed by Tukey’s test. **, P<0.01; ***, P<0.001; NS, Non significant. (PDF) [file pone.0092737.s001.pdf]

Supplementary Figure S1

A

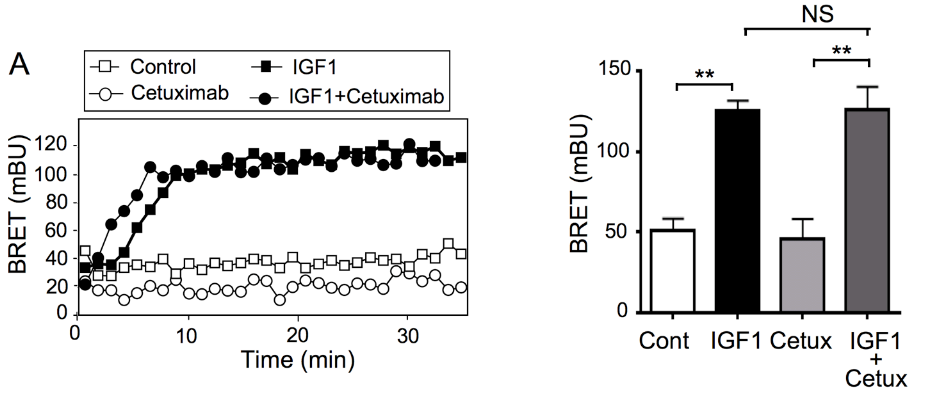

B

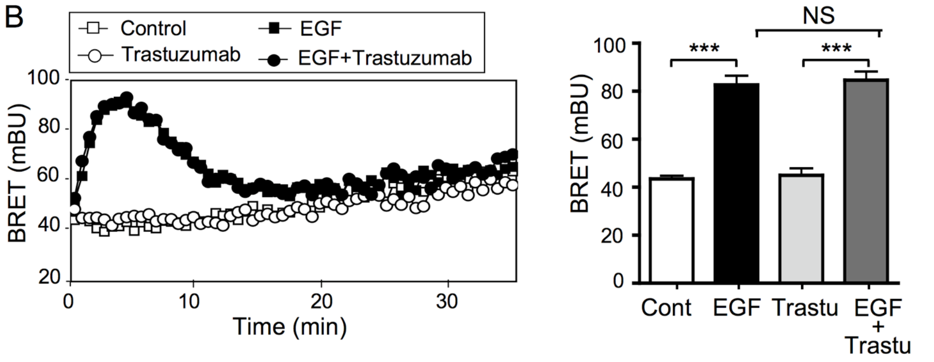

Supplementary Figure S1: Effects of anti-EGFR and anti-HER2 antibodies on IGF1 and EGF-stimulated PIP_3 production.

(A) MCF-7/B2 cells were preincubated for 1h in absence or presence of 20 ng/µl of the humanized anti-EGFR antibody Cetuximab. Cells were then stimulated with IGF1 (10 nM), and light emission acquisition started immediately. A typical real-time BRET experiment (left panel) and the mean ± SEM of BRET values at the plateau (right panel) are shown (n=3). (B) MCF-7/B2 cells were preincubated for 1h in absence or presence of 20 ng/µl of the humanized anti-HER2 antibody Trastuzumab. Cells were then stimulated with EGF (32 nM), and light emission acquisition started immediately. A typical real-time BRET experiment (left panel) and the mean ± SEM of BRET values at the peak (right panel) are shown (n=3). Statistical analysis was performed using ANOVA followed by Tukey's test. **, P< 0.01; ***, P< 0.001; NS, Non significant.
