# Supplementary material for: Development of a Human Breast-Cancer Derived Cell Line Stably Expressing a Bioluminescence Resonance Energy Transfer (BRET)-Based Phosphatidyl Inositol-3 Phosphate (PIP3) Biosensor
Source: PLoS One. 2014 Mar 19;9(3):e92737. doi: 10.1371/journal.pone.0092737 (PMC3960261; doi:10.1371/journal.pone.0092737)
Supplement: Figure S2 — Effect of inhibitors of PI3K signaling on insulin-stimulated PIP3 production in MCF-7/B2 cells. (A) MCF-7/B2 cells were preincubated for 1 h in absence or presence of 25 μM of the PI3K inhibitor LY294002. Cells were then stimulated with 10 nM insulin and light emission acquisition started immediately. A typical real-time BRET experiment (left panel) and the mean ± SEM of BRET values at the plateau (right panel) of 4 independent experiments are shown. (B, C) MCF-7/B2 cells were preincubated for 4 h in absence or presence of 10 μM of the inhibitors of Akt-PH/PIP3 interaction PIT-1 (B) and DMPIT-1 (C). Cells were then stimulated with 10 nM insulin, and light emission acquisition started immediately. Typical real-time BRET experiments (left panels) and mean ± SEM of BRET values at the plateau (right panels) of 3 to 5 independent experiments are shown. Statistical analysis was performed using ANOVA followed by Tukey’s test. *, P<0.05; **, P<0.01; ***, P<0.001; NS, Non significant. (PDF) [file pone.0092737.s002.pdf]

## Supplementary Figure S2

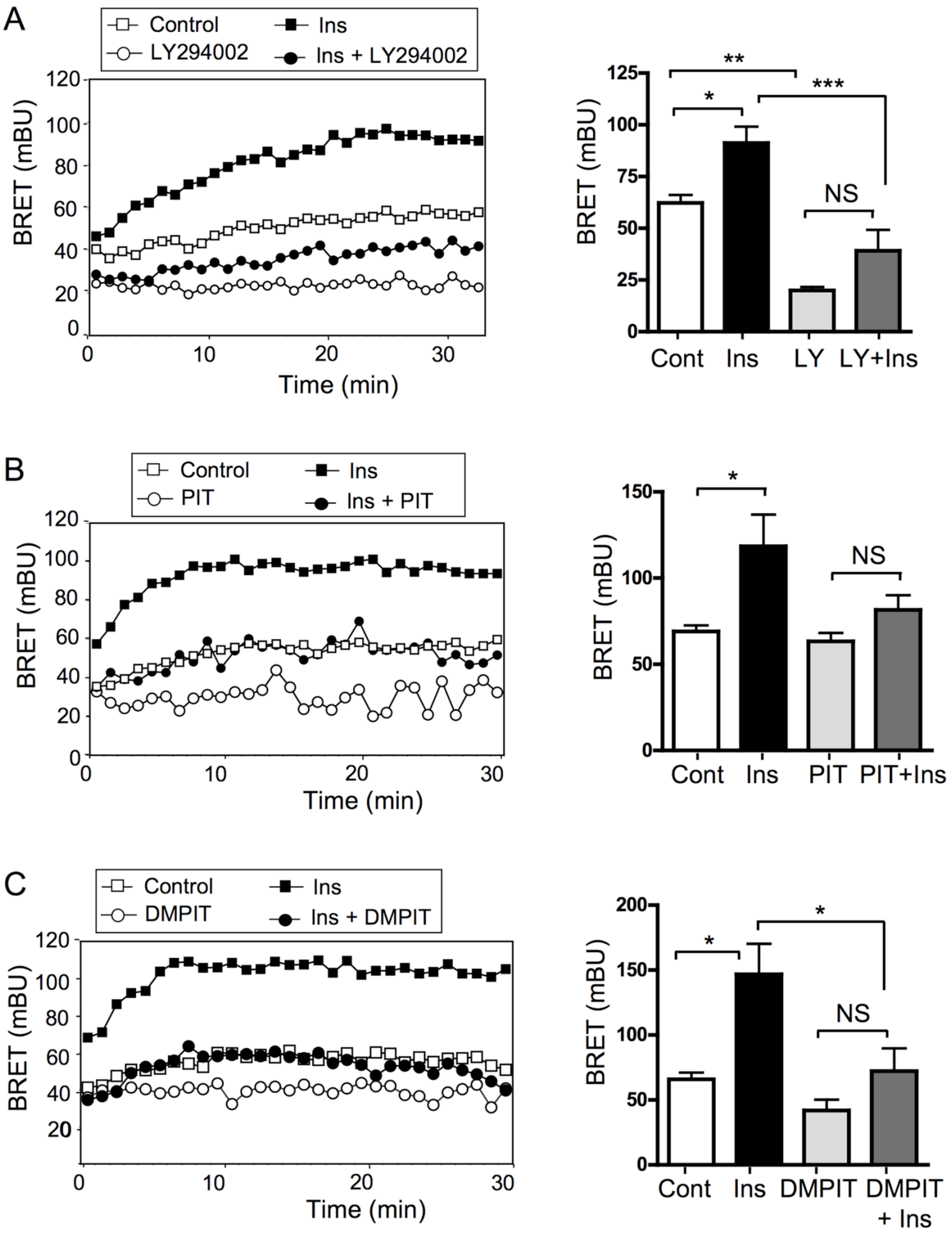


**Supplementary Figure S2: Effect of inhibitors of PI3K signaling on insulin-stimulated $PIP_3$ production in MCF-7/B2 cells.**

(A) MCF-7/B2 cells were preincubated for 1h in absence or presence of 25µM of the PI3K inhibitor LY294002. Cells were then stimulated with 10nM insulin and light emission acquisition started immediately. A typical real-time BRET experiment (left panel) and the mean ± SEM of BRET values at the plateau (right panel) of 4 independent experiments are shown. (B, C) MCF-7/B2 cells were preincubated for 4h in absence or presence of 10µM of the inhibitors of Akt-PH/ $PIP_3$ interaction PIT-1 (B) and DMPIT-1 (C). Cells were then stimulated with 10 nM insulin, and light emission acquisition started immediately. Typical real-time BRET experiments (left panels) and mean ± SEM of BRET values at the plateau (right panels) of 3 to 5 independent experiments are shown. Statistical analysis was performed using ANOVA followed by Tukey's test. *, P< 0.05; **, P< 0.01; ***, P< 0.001; NS, Non significant.
