# Supplementary material for: Development of a Human Breast-Cancer Derived Cell Line Stably Expressing a Bioluminescence Resonance Energy Transfer (BRET)-Based Phosphatidyl Inositol-3 Phosphate (PIP3) Biosensor
Source: PLoS One. 2014 Mar 19;9(3):e92737. doi: 10.1371/journal.pone.0092737 (PMC3960261; doi:10.1371/journal.pone.0092737)
Supplement: Figure S3 — Dose-dependent effect of IGFBP1 on human serum induced PIP3 production in MCF-7/B2 cells. MCF-7/B2 cells were starved overnight in culture medium containing only 0.1% FBS. Cells were then stimulated with 5% human serum that had been pre-incubated for 1 h in presence of increasing concentrations of IGFBP1. Means ± SEM of BRET values at the plateau of 4 to 7 independent experiments are shown. Statistical analysis was performed using ANOVA followed by Tukey’s test. *, P<0.05; **, P<0.01; ***, P<0.001. (PDF) [file pone.0092737.s003.pdf]

Supplementary Figure S3

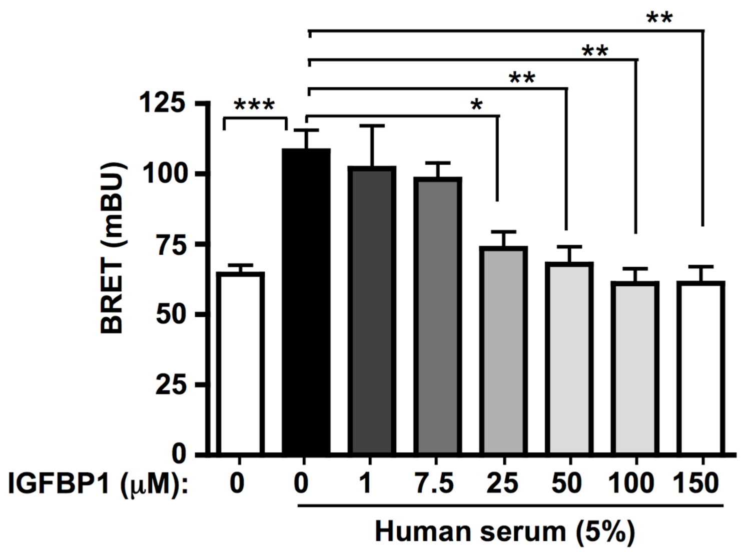

Supplementary Figure S3: Dose-dependent effect of IGFBP1 on human serum induced PIP_3 production in MCF-7/B2 cells.
MCF-7/B2 cells were starved overnight in culture medium containing only 0.1% FBS. Cells were then stimulated with 5% human serum that had been pre-incubated for 1h in presence of increasing concentrations of IGFBP1. Means ± SEM of BRET values at the plateau of 4 to 7 independent experiments are shown. Statistical analysis was performed using ANOVA followed by Tukey's test. *, P< 0.05; **, P< 0.01; ***, P< 0.001.
